# The Role of Polymorphism in the Endothelial Homeostasis and Vitamin D Metabolism Genes in the Severity of Coronary Artery Disease

**DOI:** 10.3390/biomedicines11092382

**Published:** 2023-08-25

**Authors:** Anastasia Ponasenko, Anna Sinitskaya, Maxim Sinitsky, Maria Khutornaya, Olga Barbarash

**Affiliations:** Research Institute for Complex Issues of Cardiovascular Diseases, 650002 Kemerovo, Russia; avapanass@mail.ru (A.P.);

**Keywords:** cardiovascular diseases, coronary artery disease, SYNTAX, endothelial homeostasis, vitamin D, genetic polymorphism, ELISA

## Abstract

Coronary artery disease (CAD) remains one of the leading causes of cardiovascular morbidity and mortality worldwide. The maintenance of endothelial homeostasis and vitamin D metabolism play an important role in CAD pathogenesis. This study aimed to determine the association of endothelial homeostasis and vitamin D metabolism gene polymorphism with CAD severity. A total of 224 low-risk patients (SYNTAX score ≤ 31) and 36 high-risk patients (SYNTAX score > 31) were recruited for this study. The serum level of E-, L- and P-selectins; endothelin; eNOS; 25OH; and 1.25-dihydroxy vitamin D was measured using an enzyme-linked immunosorbent assay (ELISA). Polymorphic variants in *SELE*, *SELP*, *SELPLG*, *END1*, *NOS3*, *VDR* and *GC* were analyzed using a polymerase chain reaction (PCR). We found no differences in the serum levels of the studied markers between high- and low-risk patients. Three polymorphic variants associated with CAD severity were discovered: *END1* rs3087459, *END1* rs5370 and *GC* rs2298849 in the log-additive model. Moreover, we discovered a significantly decreased serum level of 1.25-dihydroxy vitamin D in high-risk CAD patients with the A/A–A/G genotypes of the rs2228570 polymorphism of the *VDR* gene, the A/A genotype of the rs7041 polymorphism of the *GC* gene and the A/A genotype of the rs2298849 polymorphism of the *GC* gene.

## 1. Introduction

Coronary artery disease (CAD) is characterized by chronic hypoxia and an insufficient supply of nutrients to the heart resulting from the accumulation of lipids and immune cells in the subendothelial space of the coronary arteries, which is caused by atherosclerosis and atherosclerotic plaque formation [1]. CAD remains one of the leading causes of cardiovascular morbidity and mortality worldwide and, although the mortality of CAD has decreased in recent decades, it still presents a significant economic burden [2,3,4].

CAD pathogenesis is genetically determined. According to an analysis of genome-wide association studies (GWAS), 396 single-nucleotide polymorphisms (SNPs) were associated with this disease [5]. Generally, CAD-associated genes can be classified into three groups: disease-causing genes (*LDLR*, *APOB*, *PCSK9*, *CYP7A1*, *ARH* and *ABCA1*); susceptibility genes (*USF1* and *LTA*); and disease-linked genes (*ICAM2*, *PIM2*, *ECGF1*, *CXCR4*, *BL34*, *GOS8*, *ARHGAP4*, *RARA*, *RARB* and *ARRB2*) [1]. In 2011, GWAS and consortia-based studies performed in the UK, the US and Europe described 45 genes involved in CAD pathogenesis, including *SORT1*, *MIA3*, *WDR12*, *PCSK9*, *CDKN2A*, *CDKN2B*, *MRAS*, *ANRIL*, *PHACTRI1*, *PTPN11*, *ATXN2*, *CXCL12*, *SL5A3*, *SH2BS*, *LDLR*, *KCNE2* and *MRPS6* [6]. Moreover, several novel genes involved in CAD pathogenesis (*LIPA*, *PDGFB*, *ADAMTS7-MORF4L* and *KIAA1462*) were identified in South Asian patients [7].

Endothelial dysfunction caused by the violation of endothelial homeostasis is one of the most important links in CAD pathogenesis. The inflammatory activation of endothelial cells leads to the overexpression of cell adhesion molecules followed by the adhesion of mononuclear cells from the blood to the endothelium, which, in turn, is a trigger for atherosclerosis [8,9]. Endothelial cells also produce the vasodilator and vasoconstrictor molecules nitric oxide (NO) and endothelin. Imbalance in the production of these molecules leads to the loss of the vasodilatory abilities of endothelium and, ultimately, endothelial dysfunction [10,11,12,13]. 

In the last decade, vitamin D levels have been described as a novel risk factor for a number of cardiovascular diseases (CVDs), including CAD [14,15,16,17]. 25OH vitamin D can reduce CVD-associated inflammation via the stimulation of anti-inflammatory cytokine release. In kidneys, macrophages, endothelial cells and smooth muscle cells, 25OH vitamin D is metabolized into 1.25-dihydroxy vitamin D (calcitriol), which downregulates renin expression and renin-angiotensin-aldosterone system activity and reduces cardiovascular risk [18].

Despite the previously obtained results, some issues related to the genetics of CAD, especially the genetic determination of CAD severity, are still poorly investigated. Therefore, this study aimed to determine the association of endothelial homeostasis and vitamin D metabolism gene polymorphism with CAD severity.

## 2. Materials and Methods

### 2.1. Group Description

A total of 260 Caucasian patients living long-term (for at least two generations) in the Kemerovo region (Russian Federation) and hospitalized at the Research Institute for Complex Issues of Cardiovascular Diseases (Kemerovo, Russia) were recruited for this study. Stable CAD was confirmed through coronary angiography according to the guideline of the Russian Society of Cardiology on the diagnosis and treatment of stable angina. According to the SYNTAX score, 224 patients were assigned to the low-risk group (SYNTAX score ≤ 31), and 36 patients were assigned to the high-risk group (SYNTAX score > 31). Patients with cancer, autoimmune and mental and inflammatory diseases were excluded from this study. The full clinical and demographic characteristics of the patients recruited in this study are presented in Table 1.

The study design was approved by the Institutional Review Board of the Research Institute for Complex Issues of Cardiovascular Diseases (Kemerovo, Russia). All patients recruited in the presented study provided written informed consent to participate in the examination. This study complies with the Declaration of Helsinki (ethical principles for medical research involving human participants, amended in 2000) and Good Clinical Practice guidelines.

### 2.2. Enzyme-Linked Immunosorbent Assay 

Whole blood was collected from cubital vein in vacuum tubes containing coagulation activator, centrifuged for 10 min at 2500 rpm, aliquoted in 1.5 mL Eppendorf tubes and stored at −80 °C. Serum levels of E-, L- and P-selectins, endothelin, eNOS, 25OH vitamin D and 1.25-dihydroxy vitamin D were measured via enzyme-linked immunosorbent assay (ELISA) using Human sE-selectin Platinum ELISA Kit (eBioscience, San Diego, CA, USA), Human sL-selectin Platinum ELISA Kit (eBioscience), Human sP-selectin Platinum ELISA Kit (eBioscience), Endothelin-1 Quantikine ELISA Kit (R&D Systems, Minneapolis, MN, USA), Human eNOS DuoSet ELISA Kit (R&D Systems, USA), 25OH Vitamin D Total ELISA Kit (DiaSource Diagnostics, Louvain-la-Neuve, Belgium) and 1.25-Dihydroxy Vitamin D EIA Immunoassay (Immunodiagnostic Systems, East Boldon, UK) in accordance with the manufacturers’ protocols. Optical density of samples was measured using a Multiskan Sky Microplate Spectrophotometer (ThermoScientific, Waltham, MA, USA).

### 2.3. Molecular Genetic Testing

Whole blood was collected from cubital vein in vacuum tubes containing K3EDTA and stored at −80 °C. Genome DNA was extracted using the routine phenol-chloroform extraction method. 

Single-nucleotide polymorphisms (SNPs) were selected according to the following criteria: (i) location within genes involved in the maintenance of endothelial homeostasis and vitamin D metabolism; (ii) minor allele frequency > 5% in Caucasian populations; (iii) functional consequences and related studies on their role in CAD pathogenesis. Based on these criteria, thirteen SNPs in seven genes were selected: *SELE* (rs5361 and rs1805193), *SELP* (rs6136), *SELPLG* (rs2228315), *EDN1* (rs5370 and rs3087459), *NOS3* (rs1799983 and rs2070744), *VDR* (rs2228570 and rs731236) and *GC* (rs7041, rs1155563 and rs2298849) (Table 2).

Polymorphic variants in the selected genes were analyzed with allele-specific real-time polymerase chain reaction (real-time PCR) with fluorescently labeled TaqMan primers (Applied Biosystems, Waltham, MA, USA). Per analyzed sample, 10 μL of reaction mixture containing 1.25 μL of TaqMan primers (Applied Biosystems), 1 mM of dNTP (Life Technologies, Carlsbad, CA, USA), 1 U of Taq DNA polymerase (Life Technologies) and 100 ng of genome DNA template was prepared. The amplification was performed using a ViiA 7 Real-Time PCR System (Applied Biosystems) as follows: 10 min at 95 °C, 15 s at 95 °C and 60 s at 60 °C (40 cycles). The PCR quality was controlled by repeated genotyping of 10% of samples.

### 2.4. Statistical Analysis

Statistical analysis was performed using Prism 8 (version 8.4.3) software package (GraphPad Software, Boston, USA). The D’Agostino–Pearson normality test was used to verify the compliance of the obtained data with normal distribution. For quantitative data, median (m), lower (Q1) and upper (Q3) quartiles were calculated. Significant differences between groups were assessed with the Mann–Whitney *U* test and the Kruskal–Wallis *H* test. The genotyping results were analyzed using the SNPStats web tool and presented as odds ratio (OR) and 95% confidence interval (CI) calculated for dominant, recessive and log-additive inheritance models. The full description of inheritance models is presented in SNPstats tutorial (https://www.snpstats.net/tutorial.htm, accessed on 11 April 2023). The most likely inheritance model for each SNP was determined using the Akaike’s information criterion (AIC). The differences were considered statistically significant at *p* < 0.05.

## 3. Results

### 3.1. Association of Polymorphism in the Endothelial Homeostasis and Vitamin D Metabolism Genes with CAD Severity 

Three risk SNPs associated with CAD severity in the log-additive inheritance model were discovered: two variants in the maintenance of endothelial homeostasis genes—*EDN1* rs5370, OR = 2.18, 95% CI = 1.23–3.88, *p* = 0.008 and *EDN1* rs3087459, OR = 1.89, 95% CI = 1.04–3.44, *p* = 0.04; and one variant in the vitamin D metabolism genes—*GC* rs2298849 (OR = 2.26, 95% CI = 1.28–3.99, *p* = 0.006) (Table 3). Dominant and recessive inheritance models were characterized by no significant risk/protective effects on CAD severity.

### 3.2. Serum Level of Endothelial Homeostasis and Vitamin D Metabolism Markers

ELISA results revealed no significant differences in the concentration of E-, L- and P-selectins, endothelin, eNOS, 25OH vitamin D and 1.25-dihydroxy vitamin D in the serum blood obtained from the low-risk and high-risk CAD patients (Table 4).

After stratification of the patients included in the presented study according to the carried genotype, we discovered a significantly lower serum level of 1.25-dihydroxy vitamin D in the high-risk CAD patients compared with the low-risk ones, with the A/A–A/G genotypes of the rs2228570 polymorphism of the *VDR* gene (33.17 pg/mL vs. 67.47 pg/mL), the A/A genotype of the rs7041 polymorphism (12.36 pg/mL vs. 72.39 pg/mL) and the A/A genotype of the rs2298849 polymorphism (36.43 pg/mL vs. 73.24 pg/mL) of the *GC* gene (Table 5).

## 4. Discussion

The maintenance of endothelial homeostasis is critical for a normal functioning of the endothelium, which performs the most important physiological functions (transmission of different molecules into subendothelial space, regulation of hemostasis and vascular tone, prevention of platelet aggregation, etc.) [13]. The violation of endothelial homeostasis can lead to endothelial disfunction, underlying atherogenesis and its clinical manifestation—CAD [19]. Endothelial dysfunction can be defined as a loss of the anti-inflammatory, anti-thrombotic and vasodilatory abilities of endothelial cells [20].

Regulation of vascular tone is extremely important for the prevention of various cardiovascular diseases. In response to external mechanical (e.g., shear stress) and chemical (e.g., acetylcholine, bradykinin and ATP) stimuli, the endothelium produces different vasodilator (nitric oxide, prostacyclin and endothelium-derived hyperpolarization factor) and vasoconstrictor factors (thromboxane A2 and endothelin-1) that regulate the vascular tone [21,22,23]. Endothelin-1, encoded by the *EDN1* gene, is characterized by long-lasting action and remains the most important vasoconstrictor in the human cardiovascular system [24]. Endothelin-1 is continuously synthesized by endothelial cells from pre-proendothelin (preproET-1). The release of endothelin-1 is controlled by the transcription and post-transcription levels, with the implication of numerous transcription factors (AP-1, *NF*-κB, FOXO1, VezF1, HIF-1 and GATA2), physical and chemical stimuli (shear stress, hypoxia, thrombin, angiotensin II, etc.) [25,26,27]. Transforming growth factor beta (TGF-β) can be described as another regulator of endothelin-1 production in endothelial cells. It has been shown that TGF-β signaling via the ALK5/Smad3 pathway leads to an increased expression of preproET-1 [28].

The serum level of endothelin-1 has been studied as a potential risk marker for adverse cardiovascular events (atherosclerosis, CAD, arterial hypertension, myocardial infarction, heart failure, increased left atrial diameter and left ventricular mass) [29,30]. Endothelin-1 plays an important role in CAD pathogenesis via involvement in endothelial dysfunction, inflammation, atherosclerotic plaque formation, myocardial necrosis, arrhythmogenesis and, finally, left ventricular remodeling and fibrosis [31]. Endothelin-1 can induce endothelial dysfunction via the inhibition of NO pathway activity, increases in oxidative stress and inflammation and the dysregulation of glucose and lipid metabolism [32]. Moreover, endothelin-1 can inhibit the activity of endothelial NO synthase (eNOS), resulting in a decrease in NO bioavailability and NO-dependent vasorelaxation [33]. Endothelin-1 enhances ROS-induced local oxidative stress in the vascular wall via a Ras-dependent pathway [33] accompanied by local inflammation and followed by an increase in vascular permeability, leukocyte infiltration and atherosclerotic plaque formation [34,35]. Endothelin-1 can also accelerate the progression of atherosclerosis via upregulation of the mitogen-activated protein kinase pathway [36]. In addition, endothelin-1 can upregulate lipid metabolism genes and promote atherosclerotic lesions characterized by overexpression of endothelial-targeted endothelin-1 in mice; the blockage of the endothelin-A receptor leads to the restoration of endothelial function and the inhibition of atherosclerosis development in ApoE knockout mice [37,38]. 

According to molecular genetic studies, polymorphism in the *EDN1* gene is associated with the risk of CAD. It was shown that carriers of the T allele of the 5665G > T polymorphic variant, the CC and CT genotypes of the rs6458155 variant and the haplotype rs6458155–rs4145451 containing the C allele of the rs6458155 variant of the *EDN1* gene were characterized by increased CAD risk. In addition, the CT genotype of the rs6458155 variant was associated with the increased serum level of endothelin-1 compared with the TT genotype. Carriers of the homozygous variant allele of the *EDN1* gene −839T > G polymorphism were characterized by decreased CAD risk [39,40]. At the same time, there are no studies describing the role of a genetic polymorphism in the *EDN1* gene in CAD severity. In our study, we have found the associations of genetic polymorphism in the *EDN1* gene with CAD severity with a simultaneous absence of associations between genetic polymorphism and the serum level of endothelin-1. We suppose that the carriage of risk alleles of the *EDN1* gene does not lead to a quantitative change in the level of endothelin-1, but it does modify its activity, which, in turn, affects CAD progression.

25OH vitamin D is mainly synthesized endogenously from 7-dehydrocholesterol under exposure to ultraviolet radiation and is metabolized in the kidneys into their active form 1.25-dihydroxy vitamin D (calcitriol) [17,41]. Calcitriol is characterized by several systemic effects, including anti-inflammatory, anti-thrombotic and anti-atherosclerotic actions [42]. It has been shown that vitamin D deficiency is associated with a number of negative cardiovascular events including hypertension [43], myocardial injury [44] and CAD progression [45]. At the molecular level, vitamin D downregulates the *NF*-κB pathway in epicardial adipose tissue (EAT) and thereby attenuates CAD progression [45]. EAT adipocytes are deeply involved in CAD pathogenesis through the release of pro-inflammatory cytokines IL-6, IL-8 and TNF-α [46,47,48]. Membrane transporter KPNA4, expressed by EAT adipocytes, stimulates the transfer of *NF*-κB into the nucleus, where it acts as a transcription factor that upregulates the expression of pro-inflammatory cytokines [49,50]. It has been shown that the liganded 1.25-dihydroxy vitamin D receptor (VDR) can reduce atherogenesis-associated inflammatory responses via downregulating the transcription and translation of KPNA4 in EAT cells, followed by the compromised transfer of *NF*-κB into the nucleus [46]. Moreover, vitamin D and VDR can regulate NO synthesis via regulation of eNOS bioactivity in a PI3K/Akt-dependent manner and prevent oxidative stress-induced endothelial dysfunction [51].

Scientists have discovered more than 470 SNPs in the *VDR* gene, and thoroughly studied the role of four SNPs, rs2225870, rs1544410, rs7975232 and rs731236, in the pathogenesis of different diseases (cancer, diabetes, Parkinson’s disease, myocardial infarction and CAD) [52,53,54,55]. Studies have shown that genetic polymorphism in the *VDR* gene is associated with CAD risk in different populations. The rs731236 polymorphism is associated with increased CAD risk in Pakistani and Croatian populations [56,57]. At the same time, researchers from Spain and South Iran reported no correlation between this polymorphic variant and susceptibility to CAD development [58,59]. Contradictory data have been obtained for the rs1544410 polymorphism— the A allele may have both risky [60] and protective effects [59] in relation to CAD development. In the Spanish population, the rs1544410 polymorphism is not associated with the risk of CAD [58]. For the rs2225870 polymorphism, the TT genotype and CAC haplotype are associated with increased CAD risk in patients from Spain [58], but not from Croatia [57]. Moreover, this polymorphism may be predisposed to the development of premature CAD in healthy people with a family history of this disease over the next years [61]. According to the latest meta-analysis, the polymorphic variants rs2225870, rs1544410 and rs731236 are characterized by risk of CAD development, but the role of the rs7975232 polymorphism is ambiguous [62,63]. The role of genetic polymorphism in the *VDR* gene in CAD severity has been studied in only one article—the authors reported no associations between the rs1544410 and rs731236 polymorphisms with the severity of this disease [59]. In our research, we have found no associations of the rs2225870 and rs731236 polymorphisms with CAD severity, but registered significantly lower serum level of 1.25-dihydroxy vitamin D in the high-risk CAD patients with the A/A-A/G genotypes of the rs2225870 polymorphism. This polymorphic variant is known to be involved in the modulation of the response to vitamin D supplementation via regulation of calcitriol signaling [64]. 

The main transport protein of vitamin D in the plasma is vitamin D-binding protein (VDBP) encoded by the *GC* gene. The serum level of vitamin D is strongly correlated with the VDBP concentration [65]. The formation of the VDBP/25OH vitamin D complex, its filtration and reabsorption through VDR are important factors affecting the maintenance of optimal serum levels of vitamin D [66]. It was reported that the rs7041 polymorphism in the *GC* gene can be a risk factor for CAD and vascular calcification [60,67,68,69]. In the presented research, we have found that polymorphism in the *GC* gene is associated with CAD severity and decreased serum levels of calcitriol, which indicates a violation of vitamin D metabolism in carriers of risk alleles of this gene.

It should be noted that this study has limitations: the group of high-risk CAD patients is relatively small, and the studied population is homogenous. 

## 5. Conclusions

Genetic polymorphism in the endothelial homeostasis and vitamin D metabolism genes is associated with CAD severity in Caucasian patients. The obtained results can be used to assess the personalized risk of complications in CAD patients and develop appropriate early-prevention strategies in high-risk groups of patients.

## Figures and Tables

**Table 1:** Clinical and demographic characteristics of patients.

Index	Number (%)			*p*-Level
	General Group (N = 260)	Low-Risk Group (N = 224)	High-Risk Group (N = 36)	
Male	209 (80.38)	183 (81.69)	26 (72.22)	0.26
Female	51 (19.62)	41 (18.31)	10 (27.78)	0.26
Age ≤ 60 years	140 (53.85)	119 (46.88)	21 (58.33)	0.68
Age over 60 years	120 (46.15)	105 (53.23)	15 (41.67)	0.68
Angina classes III–IV	131 (50.38)	107 (47.77)	24 (66.67)	0.054
Chronic heart failure class II–III	254 (97.69)	218 (97.32)	36 (100.00)	0.40
Myocardial infarction	183 (70.38)	152 (67.86)	31 (86.11)	**0.017**
Atrial fibrillation	26 (10.00)	21 (9.38)	5 (13.89)	0.28
Arterial hypertension	248 (95.38)	213 (81.92)	35 (97.22)	0.48
Isolated coronary artery ectasia	80 (30.76)	68 (30.36)	12 (33.33)	0.42
Multifocal atherosclerosis	180 (69.24)	156 (69.64)	24 (66.67)	0.42
Stenosis of brachiocephalic arteries over 50%	141 (54.23)	121 (54.02)	20 (55.56)	0.50
Stenosis of lower limb arteries	103 (39.62)	88 (39.29)	15 (41.67)	0.46
Acute ischemic stroke	18 (6.92)	15 (6.69)	3 (8.33)	0.72
Body mass index ≤ 30	105 (40.38)	90 (40.18)	15 (41.67)	0.50
Type 2 diabetes	39 (15.00)	32 (14.29)	7 (19.44)	0.28
Impaired glucose tolerance	37 (14.23)	31(13.83)	6 (16.67)	0.40
Chronic obstructive pulmonary disease	5 (1.92)	4 (1.54)	1 (2.78)	0.52
Chronic renal failure	88 (33.84)	77 (39.29)	11 (30.56)	0.40
Chronic pyelonephritis and/or urolithiasis	83 (31.92)	72 (32.14)	11 (30.56)	0.50

**Note:** Statistically significant differences between low-risk and high-risk groups are highlighted in bold.

**Table 2:** Characteristics of the selected polymorphic variants.

Gene	Encoding Protein	Reference SNP Number	Chromosomal Position	Nucleotide Change	Variant Type
*SELE* (Selectin E)	E-Selectin	rs5361	chr1:169731919	T > G	Missense Variant Ser149Arg
		rs1805193	chr1:169733631	C > A	5 Prime UTR Variant
*SELP* (Selectin P)	P-Selectin	rs6136	chr1:169594713	T > G	Missense Variant Thr756Pro
*SELPLG* (Selectin P Ligand)	P-Selectin Glycoprotein Ligand 1	rs2228315	chr12:108624122	C > T	Missense Variant Met78Ile
*EDN1* (Endothelin 1)	Endothelin-1	rs5370	chr6:12296022	G > T	Missense Variant Lys198Asn
		rs3087459	chr6:12289406	A > C	Intron Variant
*NOS3* (Nitric Oxide Synthase 3)	Endothelial Nitric Oxide Synthase	rs1799983	chr7:150999023	T > A, G	Missense Variant Asp298Glu
		rs2070744	chr7:150992991	C > G, T	Intron Variant
*VDR* (Vitamin D Receptor)	Vitamin D3 Receptor	rs2228570	chr12:47879112	A > C, G, T	Missense Variant Met1Arg/Thr/Lys
		rs731236	chr12:47844974	A > G	Synonymous Variant Ile352Ile
*GC* (GC Vitamin D Binding Protein)	Gc-Globulin	rs7041	chr4:71752617	A > C, T	Missense Variant Asp432Glu
		rs1155563	chr4:71777771	T > A, C	Intron Variant
		rs2298849	chr4:71783134	A > G	Intron Variant

**Table 3:** Association of genetic polymorphism with CAD severity.

Gene (Reference SNP Number)	Dominant Model			Recessive Model			Log-Additive Model		
	OR (95% CI)	AIC	*p*-Value	OR (95% CI)	AIC	*p*-Value	OR (95% CI)	AIC	*p*-Value
*SELE* (rs5361)	0.84 (0.28–2.57)	213.1	0.76	0.00 (0.00–NA)	212.7	0.46	0.81 (0.28–2.32)	213	0.68
*SELE* (rs1805193)	0.93 (0.30–2.84)	213.2	0.90	0.00 (0.00–NA)	212.7	0.46	0.88 (0.31–2.51)	213.2	0.80
*SELP* (rs6136)	1.35 (0.59–3.09)	212.7	0.49	0.00 (0.00–NA)	212.4	0.37	1.21 (0.56–2.62)	213	0.63
*SELPLG* (rs2228315)	1.36 (0.48–3.85)	212.9	0.57	7.24 (0.44–119.44)	211.5	0.20	1.52 (0.62–3.74)	212.4	0.38
*EDN1* (rs5370)	1.72 (0.85–3.50)	211	0.13	9.70 (2.74–34.36)	201.7	0.001	**2.18 (1.23–3.88)**	**206.3**	**0.008**
*EDN1* (rs3087459)	1.86 (0.91–3.81)	210.4	0.09	4.21 (0.95–18.63)	210.1	0.08	**1.89 (1.04–3.44)**	**209**	**0.04**
*NOS3* (rs1799983)	0.62 (0.31–1.28)	211.6	0.20	1.08 (0.44–2.64)	213.2	0.87	0.82 (0.50–1.35)	212.6	0.43
*NOS3* (rs2070744)	0.78 (0.38–1.59)	212.8	0.50	1.08 (0.44–2.64)	213.2	0.87	0.91 (0.56–1.48)	213.1	0.71
*VDR* (rs2228570)	1.03 (0.48–2.22)	211.5	0.13	0.34 (0.10–1.19)	211.4	0.15	0.78 (0.46–1.31)	212.6	0.35
*VDR* (rs731236)	1.39 (0.65–2.98)	212.7	0.39	0.93 (0.36–2.40)	213.5	0.87	1.13 (0.69–1.86)	213.2	0.62
*GC* (rs7041)	0.68 (0.33–1.42)	211.7	0.31	1.01 (0.43–2.37)	212.8	0.32	0.85 (0.52–1.39)	212.4	0.52
*GC* (rs1155563)	0.76 (0.37–1.55)	212.2	0.45	1.02 (0.28–3.72)	212.8	0.97	0.85 (0.48–1.50)	212.4	0.56
*GC* (rs2298849)	2.11 (1.03–4.35)	208.7	0.04	7.13 (1.93–26.30)	205.9	0.005	**2.26 (1.28–3.99)**	**205.2**	**0.006**

**Note:** Statistically significant results after applying the Akaike’s information criterion (AIC) are highlighted in bold.

**Table 4:** Serum level of endothelial homeostasis and vitamin D metabolism markers in the CAD patients from low-risk and high-risk groups.

Marker	Low-Risk Group, m (Q1–Q3)	High-Risk Group, m (Q1–Q3)	*p*-Value
sE-selectin, pg/mL	24.36 (14.82–33.31)	24.33 (10.37–37.69)	0.7693
sL-selectin, pg/mL	1668 (1384–1933)	1670 (1398–2204)	0.5556
sP-selectin, pg/mL	123.9 (89.8–176.8)	165.1 (97.1–197.9)	0.1984
Endothelin, pg/mL	1.55 (1.32–2.04)	1.92 (1.48–2.47)	0.1559
eNOS, pg/mL	105.9 (61.17–160.6)	124.9 (59.32–214.9)	0.1686
25OH vitamin D, nmol/mL	16.22 (12.83–21.64)	14.28 (12.56–21.98)	0.5317
1.25-dihydroxy vitamin D, pg/mL	66.59 (32.47–102.1)	40.36 (20.01–78.96)	0.0918

**Table 5:** Serum level of endothelial homeostasis and vitamin D metabolism markers in the carriers of different polymorphic variants in the studied genes.

Gene	Reference SNP Number	Genotype	Low-Risk Group, m (Q1–Q3)	High-Risk Group, m (Q1–Q3)	*p*-Value
sE-selectin, pg/mL					
*SELE*	rs5361	T/T	24.36 (14.77–33.71)	26.77 (16.31–51.85)	0.55
		G/G-T/G	23.28 (15.68–35.32)	9.45 (8.54–10.37)	0.07
	rs1805193	C/C	24.29 (14.66–33.51)	26.77 (16.31–51.85)	0.52
		A/A-C/A	25.54 (19.21–40.96)	9.45 (8.54–10.37)	0.09
sP-selectin, pg/mL					
*SELP*	rs6136	T/T	130 (98.15–181)	175.6 (134–203.5)	0.11
		T/G-G/G	107 (81.82–130.8)	97.1 (41.7–197.9)	0.88
*SELPLG*	rs2228315	C/C	123.8 (88–170.5)	165.1 (89.6–195.7)	0.36
		C/T-T/T	138.9 (122.1–211.8)	185.9 (165–206.8)	0.53
Endothelin, pg/mL					
*EDN1*	rs3087459	A/A	1.59 (1.34–2.19)	2.083 (1.57–2.48)	0.14
		A/C-C/C	1.43 (1.27–1.79)	1.57 (1.21–1.92)	0.95
*EDN1*	rs5370	G/G	1.59(1.34–2.19)	1.71 (1.40–2.47)	0.65
		G/T-T/T	1.44 (1.30–1.76)	2.08 (1.57–2.49)	0.10
eNOS, pg/mL					
*NOS3*	rs2070744	C/C	127.5 (73.93–187.29)	104.3 (55.71–304.6)	0.69
		C/T	105.9 (63.33–168.2)	156.8 (48.92–288.9)	0.30
		T/T	100.5 (45.44–143.8)	129.9 (65.39–174.5)	0.22
	rs1799983	G/G	105.2 (47.07–165.8)	92.38 (63.13–154.3)	0.86
		T/G	105.6 (60.74–143)	134.9 (58.21–172.3)	0.27
		T/T	113.4 (73.93–167.9)	285 (42.24–459.2)	0.12
25OH vitamin D, nmol/mL					
*VDR*	rs2228570	A/A-A/G	16.51 (13.13–22.41)	14.25 (12.09–21.67)	0.29
		G/G	15.86 (11.94–21.58)	23.55 (23.55)	0.64
	rs731236	A/A	17 (13.14–23.07)	13.99 (12.56–23.55)	0.65
		A/G-G/G	16.49 (12.45–22.95)	18.44 (11.57–21.88)	0.71
*GC*	rs7041	A/A-A/C	15.97 (11.91–20.35)	15.47 (10.34–22.77)	0.94
		C/C	19.39 (15.08–26.58)	14.25 (13.1721.86)	0.09
	rs1155563	C/C-C/T	16.1 (12.31–19.31)	14.02 (9.23–21.53)	0.47
		T/T	17.79 (13.09–24.68)	14.28 (13.99–21.98)	0.50
	rs2298849	A/A	16.51 (13.16–21.31)	18.52 (13.29–23.05)	0.76
		A/G	15.98 (11.72–25.41)	14.25 (14.05–20.64)	0.76
		G/G	47.43 (9.28–85.58)	10.69 (8.12–21.98)	0.80
1.25-dihydroxy vitamin D, pg/mL					
*VDR*	rs2228570	A/A-A/G	**67.41 (35.36–86.39)**	**33.17 (12.98–68.62)**	**0.012**
		G/G	57.08 (26.98–120.1)	54.11 (40.66–78.62)	0.41
	rs731236	A/A	60 (32.47–117.2)	47.73 (25.69–78.5)	0.97
		A/G	72.38 (38.43–107.3)	49.21 (18.93–84.13)	0.14
		G/G	61.39 (28.17–91.05)	36.48 (12.39–38.65)	0.15
*GC*	rs7041	A/A	**72.39 (43–119.5)**	**12.36 (9.54–36.43)**	**0.008**
		A/C	65.89 (31.1–93.81)	51.3 (36.45–155.2)	0.69
		C/C	55.89 (32.03–110.1)	36.48 (18.93–86.34)	0.43
	rs1155563	C/C	79.77 (55.83–171.5)	44.91 (36.43–53.39)	0.17
		C/T	61.55 (32.16–85.62)	42.07 (12.78–141)	0.38
		T/T	64.77 (30.31–134.2)	38.65 (21.08–85.24)	0.29
	rs2298849	A/A	**73.24 (37.76–113)**	**36.43 (12.36–59)**	**0.010**
		A/G	57.68 (27.82–87.47)	83.14 (36.48–141)	0.35
		G/G	50.9 (28.14–302.1)	28.79 (18.93–38.65)	0.53

**Note:** Statistically significant results are highlighted in bold.

## Data Availability

The data presented in this study are available on request from the corresponding author. The data are not publicly available due to privacy statements.

## References

[1] Malakar A.K., Choudhury D., Halder B., Paul P., Uddin A., Chakraborty S. (2019). A review on coronary artery disease, its risk factors, and therapeutics. J. Cell. Physiol..

[2] Mihalina E.V., Mulerova T.A., Polikutina O.M., Ogarkov M.Y. (2019). Prevalence of coronary artery isease in the indigenous population of Gornaya Shoria (the results of epidemiological studies in 1998–2001 and 2013–2017). Complex Issues Cardiovasc. Dis..

[3] Ralapanawa U., Sivakanesan R. (2021). Epidemiology and the Magnitude of Coronary Artery Disease and Acute Coronary Syndrome: A Narrative Review. J. Epidemiol. Glob. Health.

[4] Duggan J.P., Peters A.S., Trachiotis G.D., Antevil J.L. (2022). Epidemiology of Coronary Artery Disease. Surg. Clin. N. Am..

[5] Samani N.J., Erdmann J., Hall A.S., Hengstenberg C., Mangino M., Mayer B., Dixon R.J., Meitinger T., Braund P., Wichmann H.E. (2007). Genomewide association analysis of coronary artery disease. N. Engl. J. Med..

[6] Reilly M.P., Li M., He J., Ferguson J.F., Stylianou I.M., Mehta N.N., Burnett M.S., Devaney J.M., Knouff C.W., Thompson J.R. (2011). Identification of ADAMTS7 as a novel locus for coronary atherosclerosis and association of ABO with myocardial infarction in the presence of coronary atherosclerosis: Two genome-wide association studies. Lancet.

[7] Centers for Disease Control and Prevention (CDC) (2011). Prevalence of coronary heart disease—United States, 2006–2010. Morb. Mortal. Wkly. Rep..

[8] Nafisa A., Gray S.G., Cao Y., Wang T., Xu S., Wattoo F.H., Barras M., Cohen N., Kamato D., Little P.J. (2018). Endothelial function and dysfunction: Impact of metformin. Pharmacol. Ther..

[9] Silva I.V.G., de Figueiredo R.C., Rios D.R.A. (2019). Effect of Different Classes of Antihypertensive Drugs on Endothelial Function and Inflammation. Int. J. Mol. Sci..

[10] Strohbach A., Pennewitz M., Glaubitz M., Palankar R., Gross S., Lorenz F., Materzok I., Rong A., Busch M.C., Felix S.B. (2018). The apelin receptor influences biomechanical and morphological properties of endothelial cells. J. Cell. Physiol..

[11] Herrero-Fernandez B., Gomez-Bris R., Somovilla-Crespo B., Gonzalez-Granado J.M. (2019). Immunobiology of Atherosclerosis: A Complex Net of Interactions. Int. J. Mol. Sci..

[12] Marchio P., Guerra-Ojeda S., Vila J.M., Aldasoro M., Victor V.M., Mauricio M.D. (2019). Targeting Early Atherosclerosis: A Focus on Oxidative Stress and Inflammation. Oxidative Med. Cell. Longev..

[13] Medina-Leyte D.J., Zepeda-García O., Domínguez-Pérez M., González-Garrido A., Villarreal-Molina T., Jacobo-Albavera L. (2021). Endothelial Dysfunction, Inflammation and Coronary Artery Disease: Potential Biomarkers and Promising Therapeutical Approaches. Int. J. Mol. Sci..

[14] Manousaki D., Mokry L.E., Ross S., Goltzman D., Richard J.B. (2016). Mendelian randomization studies do not support a role for vitamin D in coronary artery disease. Circ. Cardiovasc. Genet..

[15] Jorge A.J.L., Cordeiro J.R., Rosa M.L.G., Bianchi D.B.C. (2018). Vitamin D deficiency and cardiovascular diseases. Int. J. Cardiovasc. Sci..

[16] Kheiri B., Abdalla A., Osman M., Ahmed S., Hassan M., Bachuwa G. (2018). Correction to: Vitamin D deficiency and risk of cardiovascular diseases: A narrative review. Clin. Hypertens..

[17] Legarth C., Grimm D., Kruger M., Infanger M., Wehland M. (2019). Potential beneficial effects of vitamin D in coronary artery disease. Nutrients.

[18] Renke G., Starling-Soares B., Baesso T., Petronio R., Aguiar D., Paes R. (2023). Effects of Vitamin D on Cardiovascular Risk and Oxidative Stress. Nutrients.

[19] Ross R. (1999). Atherosclerosis—An inflammatory disease. N. Engl. J. Med..

[20] Bertani F., Di Francesco D., Corrado M.D., Talmon M., Fresu L.G., Boccafoschi F. (2021). Paracrine Shear-Stress-Dependent Signaling from Endothelial Cells Affects Downstream Endothelial Function and Inflammation. Int. J. Mol. Sci..

[21] Segal S.S. (2005). Regulation of blood flow in the microcirculation. Microcirculation.

[22] Sandoo A., van Zanten J.J., Metsios G.S., Carroll D., Kitas G.D. (2010). The endothelium and its role in regulating vascular tone. Open Cardiovasc. Med. J..

[23] Khaddaj Mallat R., Mathew John C., Kendrick D.J., Braun A.P. (2017). The vascular endothelium: A regulator of arterial tone and interface for the immune system. Crit. Rev. Clin. Lab. Sci..

[24] Davenport A.P., Hyndman K.A., Dhaun N., Southan C., Kohan D.E., Pollock J.S., Pollock D.M., Webb D.J., Maguire J.J. (2016). Endothelin. Pharmacol. Rev..

[25] Young A., Wu W., Sun W., Benjamin Larman H., Wang N., Li Y.S., Shyy J.Y., Chien S., García-Cardeña G. (2009). Flow activation of AMP-activated protein kinase in vascular endothelium leads to Krüppel-like factor 2 expression. Arterioscler. Thromb. Vasc. Biol..

[26] Stow L.R., Jacobs M.E., Wingo C.S., Cain B.D. (2011). Endothelin-1 gene regulation. FASEB J..

[27] Spinella F., Caprara V., Cianfrocca R., Rosanò L., Di Castro V., Garrafa E., Natali P.G., Bagnato A. (2014). The interplay between hypoxia, endothelial and melanoma cells regulates vascularization and cell motility through endothelin-1 and vascular endothelial growth factor. Carcinogenesis.

[28] Castañares C., Redondo-Horcajo M., Magán-Marchal N., ten Dijke P., Lamas S., Rodríguez-Pascual F. (2007). Signaling by ALK5 mediates TGF-beta-induced ET-1 expression in endothelial cells: A role for migration and proliferation. J. Cell Sci..

[29] Barton M., Yanagisawa M. (2019). Endothelin: 30 Years From Discovery to Therapy. Hypertension.

[30] Jankowich M., Choudhary G. (2020). Endothelin-1 levels and cardiovascular events. Trends Cardiovasc. Med..

[31] Kolettis T.M., Barton M., Langleben D., Matsumura Y. (2013). Endothelin in coronary artery disease and myocardial infarction. Cardiol. Rev..

[32] Lerman A., Holmes D.R., Bell M.R., Garratt K.N., Nishimura R.A., Burnett J.C. (1995). Endothelin in coronary endothelial dysfunction and early atherosclerosis in humans. Circulation.

[33] Iglarz M., Clozel M. (2007). Mechanisms of ET-1-induced endothelial dysfunction. J. Cardiovasc. Pharmacol..

[34] Kähler J., Ewert A., Weckmüller J., Stobbe S., Mittmann C., Köster R., Paul M., Meinertz T., Münzel T. (2001). Oxidative stress increases endothelin-1 synthesis in human coronary artery smooth muscle cells. J. Cardiovasc. Pharmacol..

[35] MacKenzie A. (2011). Endothelium-derived vasoactive agents, AT1 receptors and inflammation. Pharmacol. Ther..

[36] Piechota A., Polańczyk A., Goraca A. (2010). Role of endothelin-1 receptor blockers on hemodynamic parameters and oxidative stress. Pharmacol. Rep..

[37] Barton M., Haudenschild C.C., d’Uscio L.V., Shaw S., Münter K., Lüscher T.F. (1998). Endothelin ETA receptor blockade restores NO-mediated endothelial function and inhibits atherosclerosis in apolipoprotein E-deficient mice. Proc. Natl. Acad. Sci. USA.

[38] Simeone S.M., Li M.W., Paradis P., Schiffrin E.L. (2011). Vascular gene expression in mice overexpressing human endothelin-1 targeted to the endothelium. Physiol. Genom..

[39] Bühler K., Ufer M., Müller-Marbach A., Brinkmann U., Laule M., Stangl V., Roots I., Stangl K., Cascorbi I. (2007). Risk of coronary artery disease as influenced by variants of the human endothelin and endothelin-converting enzyme genes. Pharmacogenet. Genom..

[40] Liang L.L., Chen L., Zhou M.Y., Cai M.Y., Cheng J., Chen Y., You S.K., Chen L.B., Tang Z.B., Yang X.L. (2018). Genetic susceptibility of five tagSNPs in the endothelin-1 (*EDN1*) gene to coronary artery disease in a Chinese Han population. Biosci. Rep..

[41] Al Mheid I., Patel R.S., Tangpricha V., Quyyumi A.A. (2013). Vitamin D and cardiovascular disease: Is the evidence solid?. Eur. Heart J..

[42] Saghir Afifeh A.M., Verdoia M., Nardin M., Negro F., Viglione F., Rolla R., De Luca G. (2021). Novara Atherosclerosis Study Group (NAS). Determinants of vitamin D activation in patients with acute coronary syndromes and its correlation with inflammatory markers. Nutr. Metab. Cardiovasc. Dis..

[43] Norman P.E., Powell J.T. (2014). Vitamin D and cardiovascular disease. Circ. Res..

[44] Ahmad M.I., Chevli P.A., Li Y., Soliman E.Z. (2018). Vitamin D deficiency and electrocardiographic subclinical myocardial injury: Results from National Health and Nutrition Examination Survey-III. Clin. Cardiol..

[45] Chen S., Swier V.J., Boosani C.S., Radwan M.M., Agrawal D.K. (2016). Vitamin D Deficiency Accelerates Coronary Artery Disease Progression in Swine. Arterioscler. Thromb. Vasc. Biol..

[46] Boisvert W.A., Curtiss L.K., Terkeltaub R.A. (2000). Interleukin-8 and its receptor cxcr2 in atherosclerosis. Immunol. Res..

[47] Owen M.K., Noblet J.N., Sassoon D.J., Conteh A.M., Goodwill A.G., Tune J.D. (2014). Perivascular adipose tissue and coronary vascular disease. Arterioscler. Thromb. Vasc. Biol..

[48] Fatkhullina A.R., Peshkova I.O., Koltsova E.K. (2016). The role of cytokines in the development of atherosclerosis. Biochemistry.

[49] Sun X., Icli B., Wara A.K., Belkin N., He S., Kobzik L., Hunninghake G.M., Vera M.P., Blackwell T.S., Baron R.M. (2012). Microrna-181b regulates nf-kappab-mediated vascular inflammation. J. Clin. Investig..

[50] Leonard A., Rahman A., Fazal F. (2018). Importins alpha and beta signaling mediates endothelial cell inflammation and barrier disruption. Cell Signal..

[51] Kim D.H., Meza C.A., Clarke H., Kim J.S., Hickner R.C. (2020). Vitamin D and Endothelial Function. Nutrients.

[52] Dorsch M.P., Nemerovski C.W., Ellingrod V.L., Cowger J.A., Dyke D.B., Koelling T.M., Wu A.H., Aaronson K.D., Simpson R.U., Bleske B.E. (2014). Vitamin D receptor genetics on extracellular matrix biomarkers and hemodynamics in systolic heart failure. J. Cardiovasc. Pharmacol. Ther..

[53] Lin C.H., Chen K.H., Chen M.L., Lin H.I., Wu R.M. (2014). Vitamin D receptor genetic variants and Parkinsons disease in a Taiwanese population. Neurobiol. Aging.

[54] Rivera-Leon E.A., Palmeros-Sanchez B., Llamas-Covarrubias I.M., Fernandez S., Armendariz-Borunda J., Gonzalez-Hita M., Bastidas-Ramirez B.E., Zepeda-Moreno A., Sanchez-Enriquez S. (2015). Vitamin-D receptor gene polymorphisms (TaqI and ApaI) and circulating osteocalcin in type 2 diabetic patients and healthy subjects. Endokrynol. Pol..

[55] Ionova Z.I., Sergeeva E.G., Berkovich O.A. (2021). Genetic and epigenetic factors regulating the expression and function of the vitamin D receptor in patients with coronary artery disease. Russ. J. Cardiol..

[56] Kulsoom U., Khan A., Saghir T., Nawab S.N., Tabassum A., Fatima S., Saleem S., Zehra S. (2021). Vitamin D receptor gene polymorphism TaqI (rs731236) and its association with the susceptibility to coronary artery disease among Pakistani population. J. Gene Med..

[57] Raljević D., Peršić V., Markova-Car E., Cindrić L., Miškulin R., Žuvić M., Kraljević Pavelić S. (2021). Study of vitamin D receptor gene polymorphisms in a cohort of myocardial infarction patients with coronary artery disease. BMC Cardiovasc. Disord..

[58] González Rojo P., Pérez Ramírez C., Gálvez Navas J.M., Pineda Lancheros L.E., Rojo Tolosa S., Ramírez Tortosa M.D.C., Jiménez Morales A. (2022). Vitamin D-Related Single Nucleotide Polymorphisms as Risk Biomarker of Cardiovascular Disease. Int. J. Mol. Sci..

[59] Akhlaghi B., Firouzabadi N., Foroughinia F., Nikparvar M., Dehghani P. (2023). Impact of vitamin D receptor gene polymorphisms (TaqI and BsmI) on the incidence and severity of coronary artery disease: A report from southern Iran. BMC Cardiovasc. Disord..

[60] Kiani A., Mohamadi-Nori E., Vaisi-Raygani A., Tanhapour M., Elahi-Rad S., Bahrehmand F., Rahimi Z., Pourmotabbed T. (2019). Vitamin D-binding protein and vitamin D receptor genotypes and 25-hydroxyvitamin D levels are associated with development of aortic and mitral valve calcification and coronary artery diseases. Mol. Biol. Rep..

[61] Fronczek M., Strzelczyk J.K., Osadnik T., Biernacki K., Ostrowska Z. (2021). *VDR* Gene Polymorphisms in Healthy Individuals with Family History of Premature Coronary Artery Disease. Dis. Markers.

[62] Tabaei S., Motallebnezhad M., Tabaee S.S. (2021). Vitamin D Receptor (VDR) Gene Polymorphisms and Risk of Coronary Artery Disease (CAD): Systematic Review and Meta-analysis. Biochem. Genet..

[63] Yan X., Wei Y., Wang D., Zhao J., Zhu K., Liu Y., Tao H. (2022). Four common vitamin D receptor polymorphisms and coronary artery disease susceptibility: A trial sequential analysis. PLoS ONE.

[64] Usategui-Martín R., De Luis-Román D.A., Fernández-Gómez J.M., Ruiz-Mambrilla M., Pérez-Castrillón J.L. (2022). Vitamin D Receptor (*VDR*) Gene Polymorphisms Modify the Response to Vitamin D Supplementation: A Systematic Review and Meta-Analysis. Nutrients.

[65] Abdella N.A., Mojiminiyi O.A. (2018). Vitamin D-binding protein clearance ratio is significantly associated with glycemic status and diabetes complications in a predominantly vitamin D-deficient population. J. Diabetes Res..

[66] Thrailkill K.M., Jo C.H., Cockrell G.E., Moreau C.S., Fowlkes J.L. (2011). Enhanced excretion of vitamin D binding protein in type 1 diabetes: A role in vitamin D deficiency?. J. Clin. Endocrinol. Metab..

[67] Norman P.E., Powell J.T. (2005). Vitamin D, shedding light on the development of disease in peripheral arteries. Arterioscler. Thromb. Vasc. Biol..

[68] Lu S., Guo S., Hu F., Guo Y., Yan L., Ma W., Wang Y., Wei Y., Zhang Z., Wang Z. (2016). The associations between the polymorphisms of vitamin D receptor and coronary artery disease: A systematic review and meta-analysis. Medicine.

[69] Amadori D., Serra P., Masalu N., Pangan A., Scarpi E., Bugingo A.M., Katabalo D., Ibrahim T., Bongiovanni A., Miserocchi G. (2017). Vitamin D receptor polymorphisms or serum levels as key drivers of breast cancer development? The question of the vitamin D pathway. Oncotarget.

